# Pathogenic and Clinical Relevance of Serum IL-17A and TNF-α in Systemic Lupus Erythematosus

**DOI:** 10.3390/ijms27031244

**Published:** 2026-01-26

**Authors:** Patricia Richter, Luana Andreea Macovei, Ciprian Rezus, Alexandra Maria Burlui, Elena Rezus

**Affiliations:** 1 Department of Rheumatology and Rehabilitation, “Grigore T. Popa” University of Medicine and Pharmacy, 700115 Iasi, Romania; patricia.richter@umfiasi.ro (P.R.);; 2 I Rheumatology Clinic, Clinical Rehabilitation Hospital, 14 Pantelimon Halipa Street, 700661 Iasi, Romania; 3 Department of Internal Medicine, “Grigore T. Popa” University of Medicine and Pharmacy, 16 University Street, 700115 Iasi, Romania; ciprian.rezus@umfiasi.ro; 4 III Internal Medicine Clinic, “St. Spiridon” County Emergency Clinical Hospital, 1 Independence Boulevard, 700111 Iasi, Romania

**Keywords:** systemic lupus erythematosus, interleukin-17A, tumor necrosis factor, cytokines, inflammation, organ damage

## Abstract

Cytokines IL-17A and TNF-α have been implicated in the dysregulated immune responses that characterize SLE, with potential relevance to specific organ involvement. This study aimed to assess their serum levels in SLE patients and to explore potential correlations with clinical, biological, and immunological features, as well as with disease activity and damage scores. We conducted a cross-sectional analysis of 88 SLE patients diagnosed according to the 2012 SLICC classification criteria and 87 controls matched by sex and age. Serum IL-17A and TNF-α levels were quantified using ELISA. Clinical and laboratory data were collected, including SLEDAI for disease activity and the SLICC/ACR Damage Index for cumulative organ damage. No significant differences were observed in serum IL-17A levels between SLE patients and healthy controls, whereas serum TNF-α levels differed significantly between the two groups. Serum IL-17A levels were significantly associated with cutaneous involvement (*p* = 0.036) and the inflammatory syndrome (*p* = 0.049). TNF-α levels were also significantly elevated in patients with cutaneous manifestations (*p* = 0.050). A positive correlation was observed between TNF-α levels and cumulative organ damage, as assessed by the SLICC/ACR Damage Index (r = 0.36, *p* < 0.001; R^2^ = 0.13), and levels were particularly higher in patients with malignancies (*p* = 0.032). A positive correlation was observed between IL-17A and TNF-α levels. No significant associations were found between serum levels of IL-17A or TNF-α and demographic factors, disease activity (SLEDAI), immunological and biological markers. Both IL-17A and TNF-α were significantly associated with cutaneous involvement in SLE patients, supporting their implication in skin-related inflammatory processes. IL-17A was additionally linked to the presence of an inflammatory syndrome. TNF-α levels correlated with cumulative organ damage and were elevated in patients with malignancies, suggesting that patients with higher TNF-α accumulated significantly more irreversible organ damage over time. No meaningful associations were observed between cytokine levels and demographic characteristics, disease duration, treatment or global SLE activity.

## 1. Introduction

Systemic lupus erythematosus (SLE) is a complex autoimmune rheumatic disorder characterized by diverse clinical manifestations, in which persistent immune activation leads to cumulative tissue injury and irreversible organ damage [1,2]. Although the exact etiology and pathophysiological mechanisms of chronic inflammation remain incompletely understood, its contribution to disease onset, flares, and progression is well established [3,4].

Cytokine abnormalities are a central component that contributes to excessive inflammation and organ damage [5]. The abnormal inflammatory state in SLE is marked by dysregulated immune cell function, along with elevated circulating levels of proinflammatory cytokines such as tumor necrosis factor alpha (TNF-α) and interleukin-17A (IL-17A) [3].

In SLE, immune dysregulation is broadly mediated by T helper (Th)2 and B lymphocytes within a complex cytokine network. The hierarchical process initiated by IFN-α-activated myeloid dendritic cells promotes the differentiation and expansion of naïve T lymphocytes, leading to the development of Th1 and Th17 lymphocytes. Th1 lymphocytes produce IFN-γ, and Th17 cells secrete IL-17, IL-21, and IL-22. These proinflammatory cytokines drive B cell proliferation, plasma cell differentiation, and the production of autoantibodies. The resulting immune complexes contribute to tissue inflammation and multi-organ damage [6,7].

Regulatory T lymphocytes (Tregs) constitute another distinct subset of Th cells involved in maintaining immune tolerance by suppressing autoreactive effector T-cell responses. Alterations in Treg frequency or functional capacity have been implicated in the pathogenesis of autoimmune diseases; however, findings in SLE remain inconclusive. Tregs exert their immunomodulatory effects in part through the secretion of anti-inflammatory cytokines, particularly IL-10 and transforming growth factor-β (TGF-β) [8].

Th17 lymphocytes represent a distinct subset of T helper lymphocytes involved in the initiation and perpetuation of autoimmune processes. These cells secrete IL-17, a proinflammatory cytokine that promotes T cell priming and stimulates epithelial, endothelial, and stromal cells to produce IL-1, IL-6, TNF-α, and various chemokines, amplifying inflammatory cascades. Although IL-17 plays a physiological role in host defense against bacterial and fungal infections, its aberrant expression has been linked to several autoimmune diseases. As noted above, Th lymphocytes drive the B cell-mediated autoantibody response, and IL-17 produced by these cells contributes to SLE pathogenesis. IL-17A is the primary member of the IL-17 family, and inhibition of the IL-17A pathway has been investigated as a therapeutic strategy in SLE [9,10].

Therapeutic agents directly targeting IL-17 have demonstrated clinical benefit in several immune-mediated disorders, including psoriatic arthritis and spondyloarthritis. In contrast, data supporting the efficacy of Th17-directed therapies in SLE remain limited, with the most encouraging observations reported in the context of lupus nephritis. Overall, robust evidence to support the routine use of IL-17-targeted treatment strategies in SLE is currently lacking. Given the marked heterogeneity of SLE and the involvement of multiple immune pathways in its pathogenesis, it is improbable that blockade of a single cytokine would be universally effective across all disease manifestations. Nevertheless, selective inhibition of the Th17/IL-17 axis may prove beneficial in well-defined clinical subsets. Future studies are therefore needed to identify patient subgroups in whom Th17-targeted therapeutic approaches may be most appropriate [11].

The complexity of TNF-α biology contributes to uncertainty regarding its role in SLE, as this cytokine exerts physiological and pathological effects. In addition to its cytotoxic activity on various cell types, TNF-α promotes inflammation by regulating vascular adhesion molecules, including the upregulation of intercellular adhesion molecule-1 on endothelial cells, which facilitates leukocyte aggregation and adhesion to damaged endothelium. Moreover, through the activation of immune cells, TNF-α increases the release of inflammatory factors such as interleukins and the expression of major histocompatibility complex molecules [12,13]. TNF-α has been involved in the pathogenesis and clinical manifestations of SLE and CLE, making it a potentially therapeutic target. However, although the accumulated evidence suggests that TNF-α inhibition could be beneficial, the use of anti-TNF-α therapy in lupus remains a controversial subject [14].

Therapeutic agents targeting this cytokine have been associated with the induction of autoimmune phenomena, including the emergence of antinuclear and anti-double-stranded DNA antibodies, and in rare cases the development of an anti-TNF-α-induced lupus-like syndrome ATIL characterized by clinical features resembling SLE. Despite these observations, the ability of anti-TNF-α monoclonal antibodies to modulate inflammatory immune pathways initially raised expectations regarding their potential therapeutic utility in SLE. However, clinical studies have produced inconsistent and largely disappointing results, tempering early optimism [15,16].

The interplay between Th17-derived IL-17 and TNF-α further enhances the inflammatory milieu, especially in lupus nephritis. For example in experimental studies, stimulation of renal tubular epithelial cells with IL-17A promotes neutrophil recruitment by inducing granulocyte colony-stimulating factor (G-CSF) production. This pro-inflammatory effect is enhanced through synergistic interactions with TNF-α or IL-1β and is mediated by activation of mitogen-activated protein kinase (MAPK) signaling pathways. In parallel, IL-17 stimulation of tubular epithelial cells increases the expression of chemokines such as CXCL1, CXCL2, and CXCL8, which are chemotactic for neutrophils and monocytes. These effects are potentiated by TNF-α, contributing to the amplification of local inflammatory responses in glomerulonephritis [17].

IL-17 acts as a central amplifier of inflammatory responses by enhancing granulopoiesis, chemokine and cytokine production, and by acting synergistically with TNF-α, IFN-γ, and IL-23 [17]. These findings underscore the cooperative role of IL-17A and TNF-α in amplifying local inflammatory responses at the tissue level.

Previously, we published a review on the role of IL-17A and TNF-α in SLE. The contradictory results reported in the literature encouraged us to explore this field through an original investigation. The present study aimed to evaluate these cytokine levels in SLE patients and to determine their correlation with SLE disease activity, damage scores, clinical manifestations, laboratory parameters, and current therapies.

Patients with SLE from Eastern Europe remain underrepresented in the literature, highlighting the relevance of our manuscript. We hypothesized that higher serum levels of TNF-α and IL-17A are associated with increased disease activity and greater cumulative organ damage in LES.

## 2. Results

### 2.1. Descriptive Analysis of SLE Group and Controls

The baseline clinical characteristics of the study cohort are presented in Table 1. The analysis included 88 patients (79 females and 9 males) who fulfilled the diagnostic criteria for SLE. The age of the study population ranged between 18 and 77 years, with a mean of 51.17 ± 15.37 years and a median of 54 years. When stratified by sex, the mean age was 51.92 ± 14.80 years among female participants and 44.44 ± 19.30 years among male participants.

The duration of disease within the cohort demonstrated considerable variability, ranging from 0 to 37 years, with a mean of 10.38 ± 9.72 years and a median of 7 years.

The most frequently reported symptom at admission was arthralgia, present in 79.55% of patients, followed by fatigue in 28.41%. Cutaneous manifestations were observed in 21.59% of cases, while xerophthalmia and xerostomia were documented in 20.45% of cases. Myalgia was less commonly reported. Notably, 15 patients (17.05%) were asymptomatic.

In our study, SLEDAI scores ranged from 0 to 12, with a mean of 3.61 ± 3.23; a SLEDAI score ≥ 4 was defined as the threshold for clinical activity and was recorded in 41 patients (46.6%), while 47 patients (53.4%) had scores below this level.

To further characterize disease activity, we performed a descriptive analysis of the individual components of the SLEDAI score. The most frequent manifestations were serological: elevated anti-dsDNA antibodies were present in 39 patients (44.3%), and low complement levels in 33 patients (37.5%). Hematological abnormalities were less common, with marked thrombocytopenia (platelet count < 100,000/mm^3^) observed in 4 patients (4.6%) and marked leukopenia (leukocyte count < 3000/mm^3^) in 2 patients (2.3%). Among clinical features, rash was reported in 19 patients (21.6%) and alopecia in 12 patients (13.6%). Renal manifestations included proteinuria greater than 0.5 g/24 h in 6 patients (6.8%) and hematuria in 2 patients (2.3%). Arthritis was documented in 5 patients (5.7%), while myositis, pyuria, and mucosal ulcerations were each identified in 3 patients (3.4%). Pleurisy was rare, being observed in a single patient (1.1%). Other items of the SLEDAI score, including vasculitis, urinary casts, pericarditis, and fever greater than 38 °C, were not reported in any case.

Hydroxychloroquine was the most frequently prescribed therapy (79/88 patients, 89.8%), with a standard dose of 400 mg/day in the majority of treated patients (91.1%). Azathioprine was administered to 30/88 patients (34.1%), with available dosage data indicating a median daily dose of 100 mg (range: 50–150 mg/day). Methotrexate was used in 7/88 patients (8.0%).

Corticosteroid therapy was prescribed in 33/88 patients (37.5%). For these patients, prednisone (or equivalent) dose information was available, with a median daily dose of 10.0 mg/day.

At the time of enrollment, none of the patients were treated with leflunomide, whereas 8/88 patients (9.1%) were receiving belimumab.

The control group included 78 females and 9 males (89.7% and 10.3%), with a mean age of 51.38 ± 15.30 years.

Mean serum IL-17A levels were comparable between the control group (*n* = 87; 5.26 ± 15.12 pg/mL) and SLE patients (*n* = 88; 6.32 ± 2.70 pg/mL), with no statistically significant difference observed between groups (*t* test, *p* = 0.516).

The relationship between serum levels of IL-17A and demographic and disease-related variables was systematically explored. Correlation analyses did not reveal significant associations between IL-17A levels and age (Pearson *p* = 0.663; Spearman *p* = 0.567) or disease duration (Pearson *p* = 0.426; Spearman, *p* = 0.787). Assessment by patient gender (Mann–Whitney) revealed no significant differences in IL-17A levels (*p* = 0.146). When analyzing serum IL-17A levels according to the reason for admission, no differences were found between asymptomatic and symptomatic patients (*p* = 0.943) and no significant associations were recorded for any of the admission symptoms evaluated. Notably, patients with cutaneous involvement presented higher IL-17A levels compared to those without skin manifestations, and this difference reached statistical significance (*p* = 0.036).

Regarding disease activity, the comparison between patients with active disease (SLEDAI ≥ 4) and those with inactive disease showed no significant differences in IL-17A levels (*p* = 0.857).

Spearman’s rank correlation was used to assess the association between SLEDAI and IL-17A, given the ordinal nature and non-normal distribution of the SLEDAI score. No statistically significant correlation was observed between serum IL-17A levels and the overall SLEDAI score (Table 2).

**Figure 1:**
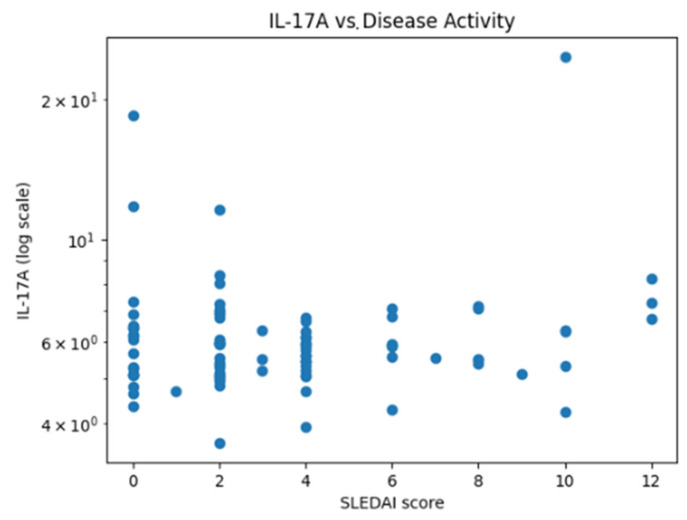
Scatter plot illustrating the relationship between serum IL-17A levels and disease activity assessed by the SLEDAI score. Each dot represents one individual patient. Serum IL-17A concentrations are displayed on a logarithmic scale to account for skewed distribution. No statistically significant monotonic association between IL-17A levels and disease activity was observed (Spearman’s rank correlation).

Due to the skewed distribution and high variability of cytokine concentrations, logarithmic transformation was applied. For IL-17A, the mean serum level was 6.32 pg/mL with a standard deviation of 2.70 pg/mL, corresponding to a coefficient of variation of approximately 43%, indicating substantial inter-individual variability. Logarithmic transformation was therefore used to improve data visualization and reduce the influence of extreme values (Figure 1).

**Figure 2:**
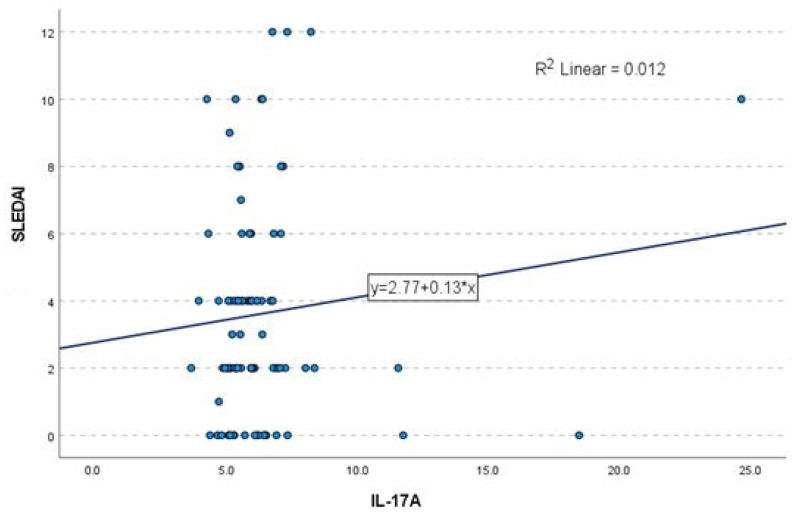
Linear regression analysis between serum IL-17A levels and SLEDAI scores in patients with SLE (*n* = 88). Each dot represents an individual patient (independent observation). No significant correlation was observed (R^2^ = 0.012), indicating that IL-17A accounted for only a minimal proportion of the variance in disease activity. Linear regression analysis was performed. No correction for multiple comparisons was applied.

Moreover, regression analysis (ANOVA) did not demonstrate a significant linear association between IL-17A levels and the global SLEDAI score (F = 1.079, *p* = 0.302). The regression coefficient for IL-17A was not statistically significant (B = 0.134, *p* = 0.302), indicating that only 1.2% of the variance in SLEDAI scores could be explained by IL-17A levels (Figure 2).

The evaluation of individual SLEDAI components by Mann–Whitney tests did not demonstrate any significant differences in IL-17A levels between patients with or without specific disease manifestations (*p* > 0.05).

Mean serum TNF-α levels were significantly higher in patients with SLE (*n* = 88; 18.29 ± 6.41 pg/mL) compared to the control group (*n* = 87; 8.57 ± 2.08 pg/mL), as demonstrated by the independent samples t-test (*p* < 0.001).

Serum TNF-α levels did not show a significant correlation with age (Pearson r = 0.027, *p* = 0.806; Spearman ρ = −0.023, *p* = 0.828). No statistically significant gender differences were observed (*p* = 0.522). Disease duration also did not correlate significantly with TNF-α concentrations. The Pearson coefficient was very weak (r = 0.033, *p* = 0.763), while Spearman’s correlation suggested a trend close to the threshold of significance (ρ = 0.195, *p* = 0.069), but this could not be confirmed in the current cohort.

When analyzed according to reasons for hospitalization, TNF-α levels were significantly higher in patients with cutaneous manifestations (*p* = 0.050), while no differences were found for other symptoms.

Disease activity assessed by SLEDAI score was analyzed as a continuous variable, as well as dichotomized (≤4 vs. >4). Comparison according to SLEDAI score showed no significant difference (Mann–Whitney, *p* = 0.861), suggesting that TNF-α does not distinguish between inactive and active disease states at this cutoff.

Spearman’s rank correlation was used to evaluate the relationship between SLEDAI and serum TNF-α levels, as SLEDAI is an ordinal, non-normally distributed measure. No statistically significant correlation was observed between serum TNF-α levels and the overall SLEDAI score (Table 3).

**Figure 3:**
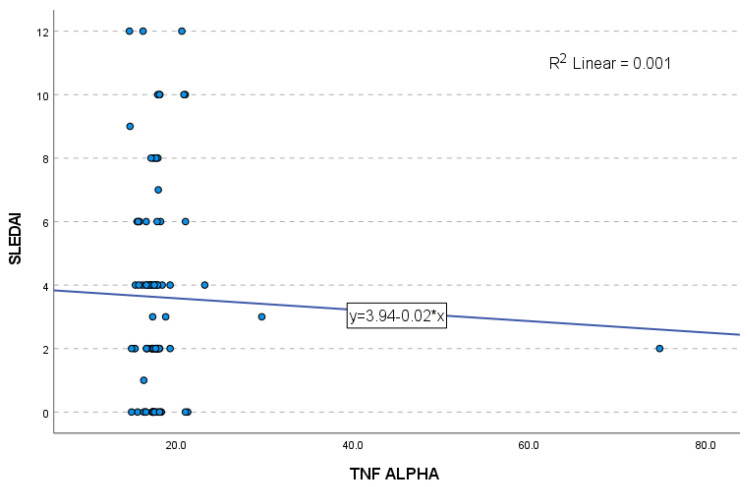
Linear regression analysis between serum TNF-α levels and SLEDAI scores in patients with SLE (*n* = 88). Each dot represents an individual patient (independent observation). No significant correlation was observed (R^2^ = 0.001), indicating that TNF-α accounted for only a negligible proportion of the variance in disease activity. As only a single comparison was conducted, no correction for multiple comparisons was required.

The linear regression model assessing serum TNF-α as a predictor of SLEDAI global score did not reach statistical significance (F = 0.109, *p* = 0.742). The coefficient of determination (R^2^ = 0.001) indicates that only 0.1% of the variance in the SLEDAI score is explained by TNF-α levels. The standardized β coefficient was very low (β = −0.036, *p* = 0.742), confirming the lack of association between TNF-α and overall SLEDAI-defined disease activity (Figure 3).

None of the individual SLEDAI manifestations (rash, alopecia, arthritis, proteinuria, thrombocytopenia, leukopenia, anti-dsDNA positivity, low complement) were associated with significant variations in TNF-α levels.

### 2.2. Irreversible Organ Damage

In our cohort, irreversible damage, quantified using the SLICC Damage Index, affected multiple organ systems (Table 4). The mean SLICC/ACR Damage Index score was 1.33 ± 1.43 (range 0–6), with a median of 1.00. When comparing patients with SLICC ≥ 1 to those with SLICC < 1, the Mann–Whitney test did not reveal significant differences in serum levels of either IL-17A (*p* = 0.858) or TNF-α (*p* = 0.929).

Ocular involvement was identified in 6.8% of patients, while central nervous system damage was documented in 13.6%. Renal impairment was present in 19.3% and pulmonary complications in 10.2% of cases. Cardiovascular complications were the most frequent, observed in 26.1% of patients. Peripheral vascular impairment occurred in 8% of cases, musculoskeletal impairment in 11.4%, and skin damage in 3.4%. Cutaneous involvement was assessed both as clinical manifestations and as cumulative irreversible damage. While cutaneous manifestations were recorded as part of the clinical presentation (Table 1), only three patients presented permanent cutaneous damage according to the SLICC/ACR Damage Index (Table 4). These patients were part of the same SLE cohort. Urogenital damage was rare (2.3%). Malignancies occurred in a limited number of SLE patients (3.4%) and included solid tumors, namely left ovarian cancer, left breast cancer, and cervical cancer. Endocrine dysfunction was documented in 10.2% of patients. According to the SLICC/ACR Damage Index, endocrine damage is limited to the presence of diabetes mellitus persisting for at least six months. No gastrointestinal organ damage was reported.

No significant association was identified between IL-17A and the SDI global score. Pearson’s correlation (r = 0.10, *p* = 0.33) did not indicate a meaningful linear relationship. Interestingly, among the organ systems evaluated by the SLICC Damage Index, a statistically significant difference in IL-17A levels was observed in patients with endocrine involvement (*p* = 0.021), whereas no significant associations were found for the other domains.

Because SLICC/ACR SDI score is a numerical count-based variable, Spearman’s rank correlation coefficient (ρ) was used for an initial non-parametric evaluation. The absence of any statistically significant association in either correlation analysis or regression models suggests that IL-17A is not a strong predictor of organ damage in this cohort of patients with SLE (Table 5).

**Figure 4:**
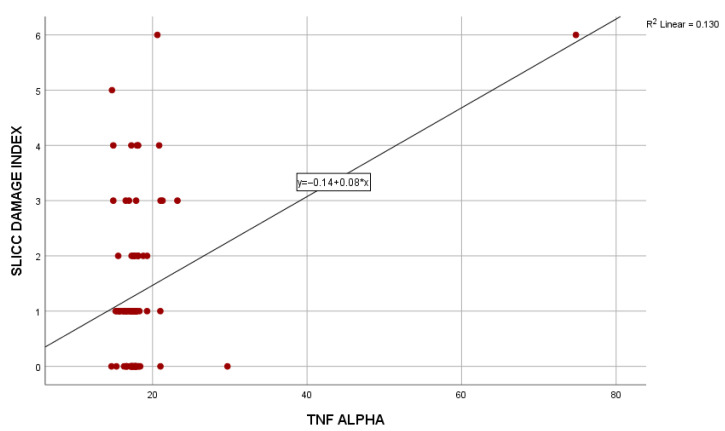
Linear regression analysis between serum TNF-α levels and cumulative organ damage assessed by SLICC/ACR Damage Index in patients with SLE (*n* = 88). A significant positive correlation was observed (R^2^ = 0.130), indicating that TNF-α accounted for 13% of the variance in cumulative organ damage. Linear regression analysis was performed. No correction for multiple comparisons was applied.

Serum TNF-α levels showed a significant positive association with cumulative organ damage as assessed by the SDI global score. Pearson’s correlation confirmed a positive relationship (r = 0.36, *p* < 0.001). Linear regression analysis demonstrated that TNF-α was a significant predictor of SDI (β = 0.36, *p* = 0.001), accounting for 13% of the variance in organ damage (R^2^ = 0.13). The regression coefficient (B = 0.08, 95% CI: 0.04–0.12) indicated that higher TNF-α values were associated with increased cumulative damage (Figure 4).

Serum TNF-α levels showed no significant associations with most organ system involvements (ocular, central nervous system, renal, pulmonary, cardiovascular, peripheral vascular, musculoskeletal, skin, urogenital, and endocrine; *p* > 0.05). A statistically significant difference was observed in patients with neoplasms, who exhibited higher TNF-α levels compared to those without malignancies (*p* = 0.032).

**Figure 5:**
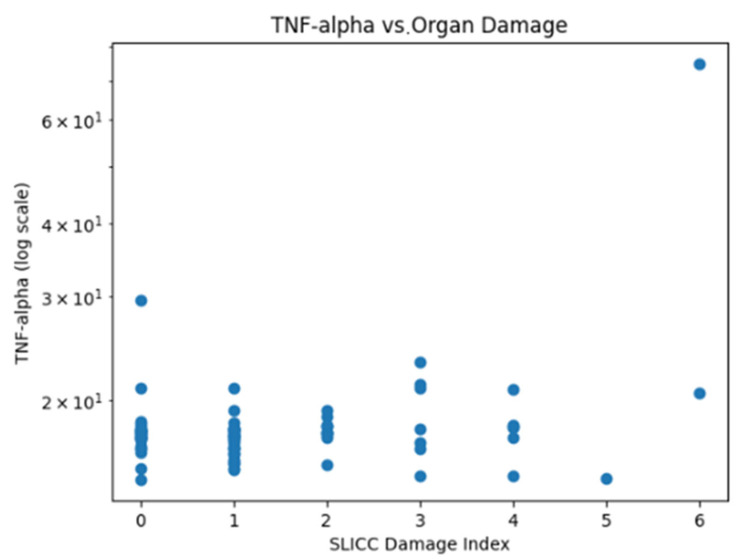
Relationship between serum TNF-α levels (log scale) and cumulative organ damage assessed by the SLICC/ACR Damage Index.

Serum TNF-α levels showed marked inter-individual variability (mean 18.29 pg/mL, SD 6.41 pg/mL; coefficient of variation ~35%). Logarithmic scaling was therefore applied for graphical visualization. The distribution of data suggests higher TNF-α levels with increasing SLICC/ACR Damage Index scores, in line with the observed statistical associations (Figure 5).

To appropriately model the relationship between serum cytokine levels and the SDI score, negative binomial regression was applied, as it represents a generalized linear model specifically designed for overdispersed count data (Table 6). In this analysis, Spearman’s correlation was statistically significant (ρ = 0.361, *p* = 0.0006), indicating a positive but moderate association between higher serum TNF-α levels and irreversible organ damage. This finding is further supported and quantified by the negative binomial regression model, which showed that for each 1 pg/mL increase in serum TNF-α concentration, the risk of accumulating an additional point in the SDI increased by approximately 2% (IRR = 1.02, 95% CI: 1.01–1.04).

**Figure 6:**
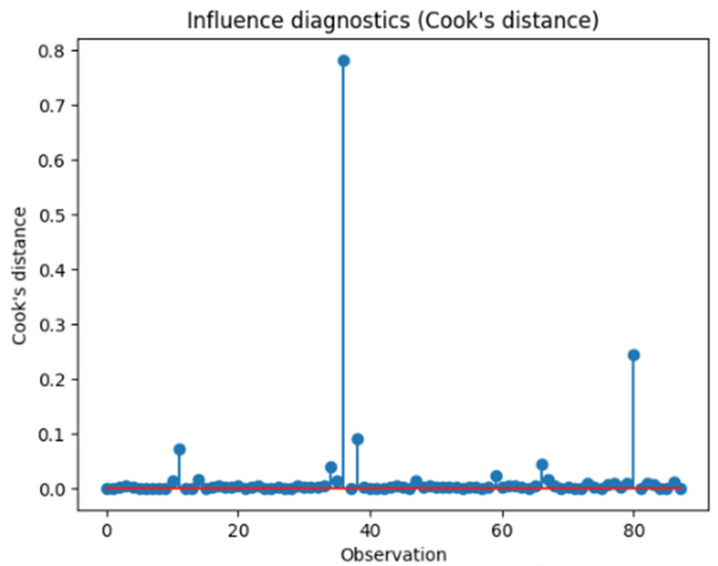
Cook’s distance influence diagnostics for the regression model evaluating the association between serum TNF-α levels and cumulative organ damage (SLICC/ACR Damage Index). The red horizontal line indicates the conventional threshold used to identify potentially influential observations.

Influence diagnostics were performed using Cook’s distance to evaluate the impact of individual observations on the regression models. The majority of observations showed low Cook’s distance values, indicating minimal influence on model estimates. Although a small number of observations exhibited higher Cook’s distance values, none exceeded the conventional threshold for undue influence, and no single data point was found to disproportionately affect the overall results (Figure 6).

### 2.3. Laboratory Parameters

Descriptive analysis showed that IL-17A levels ranged from 3.64 to 24.67 pg/mL, with a mean of 6.32 ± 2.70 pg/mL. TNF-α levels varied between 14.68 and 74.79 pg/mL, with a mean value of 18.29 ± 6.41 pg/mL. Hematological abnormalities were heterogeneous across the study group: leukopenia was observed in 23.9% of patients, anemia in 20.5%, thrombocytopenia in 9.1%, and lymphopenia in 31.8% (Table 7).

An inflammatory syndrome (elevated ESR and/or CRP) was present in 43.2% of cases. Erythrocyte sedimentation rate (ESR) ranged from 3 to 109 mm/h, with a mean value of 26.30 ± 23.08 mm/h. C-reactive protein (CRP) levels ranged from 0.01 to 2.58 mg/dL, with a mean value of 0.76 ± 0.56 mg/dL.

Biochemical changes included nitrogen retention in 23.9%, hyperglycemia in 14.8%.

Urinalysis revealed hematuria in 3.4%, leukocyturia in 21.6%, and proteinuria in 15.9%. Complement levels were decreased in 29.5% (C3) and 30.7% (C4) of patients.

Regarding immunological markers, ANA were positive in 69.2% (54/78), anti-dsDNA antibodies in 45.5% (40/88), anti-SSA in 44.4% (36/81), and anti-SSB in 24.7% (20/81). Antiphospholipid antibodies (aCL and anti-β2-GP-I) were detected in 13.2% (9/68).

**Figure 7:**
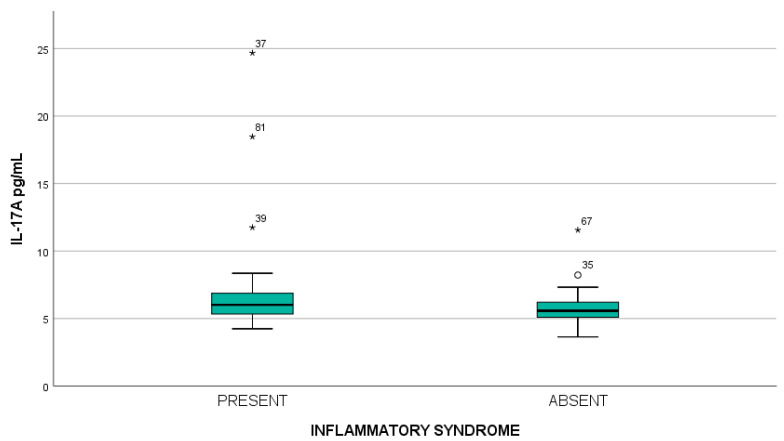
Serum IL-17A levels in SLE patients according to the presence or absence of inflammatory syndrome. Each dot represents an individual patient (independent observation; inflammatory syndrome present, *n* = 38; absent, *n* = 50). Boxes indicate the interquartile range, with the median shown as a horizontal line. Asterisks indicate extreme outliers and open circles indicate mild outliers according to boxplot convention. Statistical analysis was performed using the Mann–Whitney U test. No correction for multiple comparisons was applied.

Serum IL-17A levels did not show statistically significant differences in relation to hematological manifestations, including leukopenia, anemia, thrombocytopenia, and lymphopenia. In contrast, in patients with inflammatory syndrome, IL-17A levels were higher (7.06 ± 3.83 pg/mL; median 6.08; range 4.24–24.67) compared to those without inflammatory syndrome (5.82 ± 1.21 pg/mL; median 5.58; range 3.64–11.56), illustrated in Figure 7. This difference was confirmed by the Mann–Whitney test (mean rank 50.66 vs. 39.82, *p* = 0.049). Other biochemical alterations, such as nitrogen retention, hyperglycemia did not influence IL-17A levels. Similarly, the analysis of immunological parameters did not reveal any significant associations with serum IL-17A.

Serum TNF-α levels did not show statistically significant differences across laboratory parameters, including hematological abnormalities, biochemical markers, or immunological profiles. A trend toward higher TNF-α concentrations was noted in patients with lymphopenia (*p* = 0.091), though this did not reach statistical significance. To test the relationships among cytokines, we applied Spearman’s rank correlation. Interestingly, a positive correlation was identified between IL-17A and TNF-α (ρ = 0.274, *p* = 0.010).

### 2.4. Treatment

Antimalarial therapy with hydroxychloroquine (Plaquenil) was the baseline treatment, administered to 89.8% of patients, with a standard dose of 400 mg/day in the majority of treated cases (91.1%). Among immunosuppressants, azathioprine was the most frequently used (34.1%), with available dosage data indicating a median daily dose of 100 mg (range: 50–150 mg/day). Methotrexate was used in 7/88 patients (8.0%), while mycophenolate mofetil was prescribed in a smaller proportion of cases (4.5%). Cyclophosphamide (3.4%) and cyclosporine (1.1%) were prescribed in isolated cases. The limited number of patients receiving cyclophosphamide or cyclosporine did not permit a precise statistical analysis. therapy was documented in 37.5% of the study population. For these individuals, prednisone (or equivalent) dose information was available, with a median daily dose of 10.0 mg/day. At the time of enrollment, none of the patients were receiving leflunomide, whereas biologic therapy with belimumab was ongoing in 8/88 patients (9.1%).

Serum IL-17A levels were analyzed across therapeutic subgroups. Patients receiving hydroxychloroquine showed significantly higher IL-17A levels compared to those not on treatment (*p* = 0.021). IL-17A did not show significant variations between patients on 200 mg/day and those treated with 400 mg/day (*p* = 0.561). However, the result should be interpreted with consideration of potential biases, given the small size of the group of patients not receiving Plaquenil (*n* = 9). Corticosteroids, methotrexate, azathioprine, mycophenolate mofetil, and belimumab did not significantly influence IL-17A levels.

When IL-17A levels were dichotomized according to the cohort mean, no significant associations were observed between IL-17A category and treatment exposure. The distribution of lower versus higher IL-17A levels did not differ significantly according to hyroycholoquine use (Pearson χ^2^ = 1.98, *p* = 0.159; Fisher’s exact test *p* = 0.262), corticosteroid therapy (χ^2^ = 0.50, *p* = 0.478; Fisher’s exact test *p* = 0.489), or azathioprine treatment (χ^2^ = 0.05, *p* = 0.826; Fisher’s exact test *p* = 0.815). Similarly, no significant association was identified for belimumab exposure (Fisher’s exact test *p* = 0.259), although this analysis should be interpreted as exploratory due to the small number of treated patients.

Serum TNF-α levels were not significantly affected by corticosteroids, hydroxychloroquine, or other immunosuppressive and biologic therapies in the analyzed cohort.

When TNF-α levels were dichotomized according to the cohort mean, a significant association was observed with corticosteroid therapy, with patients receiving corticosteroids showing a lower proportion of higher TNF-α levels compared to those not receiving corticosteroids (χ^2^ = 5.10, *p* = 0.024; Fisher’s exact test *p* = 0.035). In contrast, no significant associations were identified between TNF-α category and treatment with Plaquenil (Fisher’s exact test *p* = 0.151), azathioprine (Fisher’s exact test *p* = 1.000), or belimumab (Fisher’s exact test *p* = 0.608), the latter analysis being interpreted as exploratory due to the small number of belimumab-treated patients.

## 3. Discussion

### Overview of the Main Findings

In the present study, serum TNF-α and IL-17A levels were evaluated in relation to disease activity, cumulative organ damage, clinical manifestations, laboratory parameters, and current therapies in SLE patients, and were additionally compared with healthy controls. TNF-α levels were significantly higher in SLE patients than in controls and were significantly associated with cumulative organ damage and cutaneous involvement, whereas no significant differences were observed between patients with active and inactive disease. In contrast, IL-17A levels did not differ significantly between patients and controls and were not associated with global disease activity or cumulative damage, but showed significant associations with cutaneous manifestations and inflammatory markers.

(a) Comparison with prior studies

SLE patients generally present higher circulating concentrations of TNF-α than healthy individuals [13,18,19,20,21,22,23], an important finding that was also observed in the present study.

The association between TNF-α concentrations and clinical features of SLE appears to be complex. While some investigations linked elevated TNF-α with periods of active disease [13,19,22,23,24,25,26], other studies did not demonstrate a correlation between TNF-α levels and disease activity [27,28,29,30]. In our cohort, serum TNF-α levels were similar in patients with inactive and active SLE, with no statistically significant differences between the two groups.

Elevated TNF-α has been documented not only in circulation but also locally within inflamed renal tissue of SLE patients [22,23,31]. Beyond lupus nephritis, increased TNF-α expression has also been reported in association with vasculitis [18] and with neuropsychiatric features such as depressive symptoms [32].

Previous longitudinal studies showed that patients with elevated baseline TNF-α levels accumulated significantly more irreversible organ damage over time [22]. In line with these findings, our analysis revealed a significant positive association between serum TNF-α concentrations and SLICC/ACR Damage Index scores, suggesting a potential role of this cytokine in damage accrual among SLE patients.

While some authors reported associations between anti-dsDNA antibodies or complement levels and TNF-α levels, our immunological analyses did not confirm this relationship [13,25]. Similarly, Moreno-Torres et al. did not observe significant correlations between TNF-α and anti-dsDNA antibodies [33].

Moreover, the literature does not consistently report significant correlations between TNF-α concentrations and therapeutic regimen, and our cohort showed similar findings [22,33].

Regarding IL-17, serum levels are elevated in patients with SLE compared to the general population [8,10,34,35,36,37,38,39,40,41,42,43,44,45], serum IL-17A levels were comparable between patients with SLE and healthy controls in the present study.

Some studies have reported an association between IL-17 levels and disease activity [8,9,10,35,36,39,41,44,46], whereas others have not confirmed this relationship [34,37,38,40,47,48,49,50,51,52]. Our findings align with those studies that did not observe an association between IL-17 levels and the SLEDAI score.

In our cohort, IL-17A levels did not show significant correlations with age or disease duration, aligning with previous studies that also reported no association between IL-17 and these variables [45,48].

Previous studies have demonstrated higher IL-17 levels in pediatric SLE patients with cutaneous and hematological involvement [44]; significantly elevated levels have also been reported in SLE patients with CNS disease [40]. Moreover, higher IL-17 levels have been described in lupus nephritis compared with SLE without renal involvement [41]. Our findings further support these observations, as we also identified elevated IL-17 levels in association with cutaneous manifestations.

We also found no correlation between serum IL-17A levels and cumulative organ damage, as measured by the SLICC Damage Index, which is consistent with findings from other studies [9,40,48,53].

In our cohort, serum IL-17A levels were significantly associated with the presence of endocrine manifestations. Although data regarding the role of IL-17A in endocrine involvement in SLE are limited, this finding warrants further investigation in larger and more homogeneous patient populations.

Our study demonstrated a significant association between IL-17A levels and the presence of an inflammatory syndrome, aligning with existing literature data that identify a positive correlation between IL-17A and inflammatory markers, such as high-sensitivity CRP [9]. Some studies have reported an association between serum IL-17 levels and anti-dsDNA antibodies [9,35], whereas others did not confirm this finding [45,51]; similarly, no correlation was observed in our cohort.

Studies reported no significant correlations between IL-17 levels and treatment parameters [8,34]. Tanasescu et al. reported no correlation between IL-17A and corticosteroid or hydroxychloroquine therapy [37]; in our study, IL-17 levels correlated with hydroxychloroquine therapy. However, this result should be carefully interpreted, as the subgroup distribution was unbalanced, with only nine patients not receiving hydroxychloroquine treatment.

Variability in study findings may reflect differences in cohort size, ethnic background, participants’ age, assay sensitivity for IL-17 and TNF, treatment regimens involving immunosuppressive agents, and disease duration.

(b) Biological interpretation

Within the disease process, TNF-α exerts a paradoxical influence: while it amplifies inflammation and contributes to organ injury, it can also exert regulatory functions that restrain autoimmune responses under certain conditions [14,16,54].

In a murine model, IgG-induced skin inflammation was significantly reduced in TNFR1-deficient mice, but not in those lacking TNFR2, highlighting the role of the TNF-α/TNFR1 axis in cutaneous inflammation. [55]. Consistently, a recent study investigating acute cutaneous lupus erythematosus (ACLE) in the context of SLE has identified TNF-α as a potential biomarker and independent risk factor for ACLE. The authors emphasized the close link between ACLE and systemic disease activity and proposed that elevated TNF-α levels may predict the development of cutaneous involvement, offering a potential target for early intervention [56]. In this context, the observed association between elevated TNF-α levels and cutaneous lesions in our cohort aligns with existing evidence.

The risk of malignancy in SLE is of particular concern, as it may be influenced both by the underlying immune and genetic mechanisms of the disease and by the immunosuppressive therapies used in its treatment. Our understanding of these risk factors and their implications for treating SLE and screening for malignancy is still evolving [57,58]. Therefore, the significantly elevated TNF-α levels observed in our patients with malignancies may reflect complex immunological mechanisms.

In contrast to TNF-α, IL-17A appears to exert predominantly context-dependent and tissue-specific effects in SLE. The absence of a significant difference in serum IL-17A levels between patients with SLE and controls may be related to the high proportion of patients receiving immunosuppressive therapy and exhibiting low disease activity at the time of assessment, factors that could influence circulating cytokine levels.

Elevated serum IL-17A levels have been described in patients with different types of lupus, such as SLE, discoid lupus erythematosus (DLE), and subacute cutaneous lupus erythematosus (SCLE) [6,37,59,60]. IL-17A expression is increased in most lupus skin lesions and correlates positively with interferon-α expression, supporting the hypothesis of a shared pathogenic mechanism in cutaneous lupus erythematosus [61]. Given the markedly increased IL-17 expression observed in patients with cutaneous lupus erythematosus (CLE) and SLE, anti-IL-17 therapies have been suggested as potentially effective for treating CLE lesions [62]. These observations may explain the observed significant association between IL-17A levels and cutaneous involvement in our cohort. In the present study, serum IL-17A levels were significantly associated with endocrine damage, which in the SLICC/ACR Damage Index is defined by the presence of diabetes mellitus. This finding is consistent with accumulating evidence indicating that chronic hyperglycemia in type 2 diabetes is accompanied by systemic low-grade inflammation and activation of innate and adaptive immune responses. Previous studies have shown increased circulating Th17 cells and elevated serum IL-17A levels in patients with type 2 diabetes. The IL-23/IL-17 axis has been implicated in inflammatory processes contributing to insulin resistance [63,64].

(c) Limitations and future research directions

This study also has limitations that should be acknowledged. TNF-α and IL-17A showed no statistically significant associations with most organ system involvements (ocular, central nervous system, renal, pulmonary, cardiovascular, peripheral vascular, musculoskeletal, skin, urogenital, and endocrine; *p* > 0.05). However, this finding should not be interpreted as evidence of biological independence. The relatively limited sample size, particularly within specific clinical subgroups, may have reduced the statistical power of the analyses. In particular, subgroup comparisons according to disease activity status (active vs. inactive SLE) and organ involvement may have been underpowered to detect small-to-moderate effects, raising the possibility of a type II error.

The small number of patients within SLE subgroups limited the statistical power of the comparative analyses. Although adequate for exploratory correlation and regression analyses in the overall cohort, the study may have been underpowered to detect small-to-moderate associations, particularly in subgroup analyses stratified by disease activity status (active vs. inactive disease) or specific organ involvement. Larger, adequately powered studies are warranted to confirm these observations.

Another limitation is the lack of multivariable adjustment for potential confounders such as age, sex, disease duration, treatment exposure, comorbidities, and disease activity. Given the relatively limited sample size and the number of covariates of interest, the application of fully adjusted multivariable models would have carried a substantial risk of overfitting and unstable estimates. Therefore, the analyses were intentionally restricted to univariable and stratified tests.

The absence of significant differences in serum IL-17A concentrations between LES patients and controls may be explained by cohort characteristics at the time of evaluation. Most patients were receiving immunosuppressive therapy, and disease activity was predominantly low, conditions known to attenuate circulating proinflammatory cytokine levels. Therefore, the utility of serum IL-17A and TNF as biomarkers of disease activity or flare prediction may be limited in cohorts characterized by low disease activity and ongoing immunosuppressive treatment. The lack of detailed clinical characterization of the healthy control group also represents a limitation of this study and precluded subgroup-matched comparisons for IL-17A analyses.

## 4. Materials and Methods

### 4.1. Study Population

This study enrolled 88 individuals with a confirmed diagnosis of SLE, who were monitored at the Ist Rheumatology Clinic of the Clinical Rehabilitation Hospital in Iasi between July and November 2022. The diagnosis was established according to the 2012 Systemic Lupus International Collaborating Clinics (SLICC) classification criteria [65]. Exclusion criteria included the presence of overlap syndromes, active infections, cognitive or communication impairments, and refusal to participate. All participants underwent a complete physical examination. Clinical characteristics, disease activity, and laboratory parameters were recorded at the time of evaluation. Ongoing medication was also documented. The activity of SLE was evaluated using the SLE Disease Activity Index (SLEDAI) [66,67]. At the same time, cumulative organ damage was assessed with the Systemic Lupus International Collaborating Clinics (SLICC) Damage Index [68]. SLE-related immunological and laboratory parameters were assessed, including antinuclear antibodies, anti-dsDNA antibodies, anti-SS-A and anti-SS-B antibodies, antiphospholipid antibodies, complement fractions (C3 and C4), erythrocyte sedimentation rate, C-reactive protein, together with complete blood count, and standard liver and renal function tests. In the present cohort, hyperglycemia was defined as a fasting blood glucose value > 105 mg/dL. This finding may be observed in patients with established diabetes mellitus as well as in individuals who do not fulfill diagnostic criteria for diabetes.

A control group of 87 age- and sex-matched healthy individuals was included for comparative analysis.

### 4.2. Cytokine Quantification

Serum IL-17A concentrations were quantified using a commercially available ELISA kit (BioVendor, RAF039R, Brno, Czechia) according to the manufacturer’s protocol.

To ensure analytical reliability of the ELISA measurements, assay performance characteristics were explicitly reported, including the limit of detection (LOD), intra-assay and inter-assay coefficients of variation, as well as standardized sample handling and storage conditions. The assay is based on a sandwich ELISA principle, employing a monoclonal anti-human IL-17A antibody immobilized on microtiter wells for antigen capture, followed by detection with a biotin-conjugated anti-human IL-17A antibody and streptavidin-HRP. Blood samples were collected by venipuncture, and serum was separated by centrifugation, aliquoted, and stored at −80 °C until analysis. Prior to measurement, frozen samples were thawed at room temperature and mixed gently to ensure homogeneity. Each sample was tested in duplicate. Standards were prepared by serial twofold dilutions ranging from 100.0 pg/mL to 1.6 pg/mL. Absorbance was read at 450 nm using a microplate reader, and IL-17A concentrations were interpolated from the standard curve and corrected for the dilution factor (×2). The assay’s sensitivity, defined as the lower limit of detection (LOD) was 0.5 pg/mL. The reported intra- and inter-assay coefficients of variation were 7.1% and 9.1%, respectively, indicating good assay precision.

Serum TNF-α levels were measured using a commercially available ELISA kit (BioVendor, RAF128R) according to the manufacturer’s instructions. The sandwich ELISA utilizes monoclonal antibodies specific for human TNF-α. Standard venipuncture techniques were used to collect venous blood, and sera were obtained by centrifugation and immediately stored at −80 °C until testing. All samples were assayed in duplicate. Standards, blanks, and serum samples diluted 1:2 with Sample Diluent were added to wells pre-coated with anti-human TNF-α antibodies and incubated with a biotin-conjugated detection antibody. After washing, Streptavidin-HRP was applied, followed by addition of the substrate solution. The reaction was stopped with phosphoric acid, and absorbance was read at 450 nm (reference 620 nm). A standard curve was generated from serial dilutions of recombinant TNF-α ranging from 7.8 to 500 pg/mL. Final concentrations were interpolated from the curve and corrected for the dilution factor (×2). The assay’s LOD was 2.3 pg/mL, with intra- and inter-assay coefficients of variation of 6% and 7.4%, respectively.

### 4.3. Statistical Analysis

Data analysis was performed using IBM SPSS Statistics, version 26.0. Continuous variables were summarized as minimum, maximum, and mean ± standard deviation, or as median with interquartile range (IQR). Absolute numbers and percentages described categorical variables.

Comparisons between groups were performed with the Mann–Whitney U test for non-parametric data. Correlations were examined using Pearson’s or Spearman’s rank coefficients, depending on variable distribution. Specifically, associations between serum IL-17A and TNF-α concentrations and disease activity or damage indices were evaluated using Spearman’s rank correlation coefficient (ρ), given the non-normal distribution and ordinal nature of SLEDAI and SLICC/ACR Damage Index (SDI) scores. In addition, given the count-based nature of the SDI and the presence of overdispersion, associations between SDI and serum cytokine concentrations were further examined using negative binomial regression, with effect estimates reported as incidence rate ratios (IRRs) and corresponding 95% confidence intervals.

To investigate potential predictors of IL-17A or TNF-α concentrations, simple linear regression models were applied. A two-tailed *p*-value < 0.05 was considered statistically significant.

Due to the skewed distribution and inter-individual variability of cytokine concentrations, logarithmic transformation was applied for graphical visualization purposes. Influence diagnostics were performed for regression analyses using Cook’s distance to assess the impact of individual observations on model estimates. Axis scaling in figures was adjusted to improve data interpretability.

## 5. Conclusions

In our cohort of SLE patients, serum levels of IL-17 and TNF-α were correlated with the presence of cutaneous lesions, supporting their role in the underlying inflammatory mechanisms of skin manifestations. TNF-α was associated with the SDI, confirming a significant relationship with the accumulation of long-term organ damage. IL-17A levels were higher in patients with inflammatory syndrome. No significant associations were found between these cytokines and demographic factors, global disease activity, hematological or immunological markers. Serum IL-17A levels were comparable between patients with SLE and the control group, whereas serum TNF-α levels were significantly higher in SLE patients. These findings contribute to a better understanding of cytokine-driven mechanisms in SLE and highlight the relevance of studying underrepresented populations.

## Figures and Tables

**Table 1:** Baseline demographic and clinical characteristics of SLE patients and their association with serum IL-17A and TNF-α levels.

Parameter	Patients with SLE (*n* = 88)/Number (%)	IL-17A, *p* (2-Tailed)	TNF-α, *p* (2-Tailed)
Demographics			
Female	79 (89.8)	0.146	0.522
Male	9 (10.2)		
Symptoms at admission			
Arthralgia	70 (79.6)	0.967	0.656
Fatigue	25 (28.4)	0.238	0.485
Cutaneous manifestations	19 (21.6)	0.036	0.050
Xerophthalmia/Xerostomia	18 (20.5)	0.482	0.702
Asymptomatic	15 (17.1)	0.943	0.329
Disease activity (SLEDAI)			
SLEDAI ≤ 4	47 (53.4)	0.857	0.861
SLEDAI > 4	41 (46.6)		
SLEDAI characteristics			
Rash	19 (21.6)	0.294	0.726
Alopecia	12 (13.6)	0.865	0.841
Arthritis	5 (5.7)	0.499	0.780
Proteinuria (>0.5 g/24 h)	6 (6.8)	0.574	0.685
Thrombocytopenia (<100,000/mm^3^)	4 (4.6)	0.133	0.596
Leukopenia (<3000/mm^3^)	2 (2.3)	0.823	0.779
Anti-dsDNA antibodies	39 (44.3)	0.983	0.837
Low complement	33 (37.5)	0.942	0.921

Anti-dsDNA = antibodies directed against double-stranded DNA; SLEDAI = Systemic Lupus Erythematosus Disease Activity Index.

**Table 2:** Spearman’s correlation between IL-17A and SLEDAI.

Parameter	Spearman’s Correlation Coefficient (ρ)	*p*-Value	Interpretation
IL-17A	0.058	0.590	No statistically significant correlation was observed

**Table 3:** Spearman’s correlation between TNF-α and SLEDAI.

Parameter	Spearman’s Correlation Coefficient (ρ)	*p*-Value	Interpretation
TNF-α	0.100	0.354	No statistically significant correlation was observed

**Table 4:** Irreversible organ damage in patients with SLE (*n* = 88) and its association with serum IL-17A and TNF-α levels.

Organ System	Patients with SLE (*n* = 88)/Number (%)	IL-17A, *p* (2-Tailed)	TNF-α, *p* (2-Tailed)
Ocular	6 (6.8)	0.974	0.082
Central nervous system	12 (13.6)	0.169	0.166
Renal	17 (19.3)	0.845	0.916
Pulmonary	9 (10.2)	0.831	0.940
Cardiovascular	23 (26.1)	0.846	0.375
Peripheral vascular	7 (8.0)	0.392	0.811
Musculoskeletal	10 (11.4)	0.159	0.152
Skin	3 (3.4)	0.654	0.827
Urogenital	2 (2.3)	0.240	0.801
Neoplasms	3 (3.4)	0.285	0.032
Endocrine	9 (10.2)	0.021	0.820

**Table 5:** Associations between IL-17A and cumulative damage index.

Parameter	Coefficient (ρ/IRR)	95% CI	*p*-Value
IL-17A	ρ = 0.095	–	0.378

**Table 6:** Associations between TNF-α levels and cumulative damage index.

Parameter/Test	Biological Marker	Coefficient (ρ/IRR)	95% CI	*p*-Value
TNF-α/Spearman correlation	ρ = 0.361	–	0.0006	Statistically significant moderate positive correlation
TNF-α/Negative binomial regression	IRR = 1.02	1.01–1.04	0.003	TNF-α is a significant predictor of cumulative organ damage

**Table 7:** Baseline biological, immunological, and therapeutic characteristics in patients with SLE and their association with serum IL-17A and TNF-α levels.

Parameter	Patients with SLE (*n* = 88)/Number (%)	IL-17A, *p* (2-Tailed)	TNF-α, *p* (2-Tailed)
**Laboratory parameters**			
Leukopenia	21/88 (23.9)	0.685	0.957
Deficiency anemia	18/88 (20.5)	0.959	0.291
Thrombocytopenia	8/88 (9.1)	0.622	0.867
Lymphopenia	28/88 (31.8)	0.165	0.091
Inflammatory syndrome	38/88 (43.2)	0.049	0.119
Nitrogen retention	21/88 (23.9)	0.788	0.965
Hyperglycemia	13/88 (14.8)	0.855	0.846
Low C3	26/88 (29.5)	0.708	0.522
Low C4	27/88 (30.7)	0.910	0.939
ANA	54/78 (69.2)	0.251	0.164
Anti-dsDNA	40/88 (45.5)	0.913	0.987
Anti-SSA	36/81 (44.4)	0.932	0.527
Anti-SSB	20/81 (24.7)	0.562	0.930
Antiphospholipid antibodies	9/68 (13.2)	0.196	0.385
**Treatment**			
Hydroxychloroquine	79/88 (89.8)	0.021	0.374
Azathioprine	30/88 (34.1)	0.692	0.860
Methotrexate	7/88 (8.0)	0.660	0.331
Mycophenolate mofetil	4/88 (4.5)	0.085	0.555
Belimumab	8/88 (9.1)	0.234	0.722
Corticosteroids	33/88 (37.5)	0.839	0.472

ANA = antinuclear antibody; Anti-dsDNA = antibodies directed against double-stranded DNA.

## Data Availability

The original contributions presented in this study are included in the article. Further inquiries can be directed to the corresponding authors.

## Abbreviations

The following abbreviations are used in this manuscript:

CRP	C-reactive protein
ESR	Erythrocyte sedimentation rate
aCL	Anticardiolipin antibodies
anti-β2-GP-I	Anti-beta-2 glycoprotein I antibodies
